# Extended interactive voice response telephony (IVR) for relapse prevention after smoking cessation using varenicline and IVR: a pilot study

**DOI:** 10.1186/1471-2458-13-824

**Published:** 2013-09-10

**Authors:** Bonnie McNaughton, Jiri Frohlich, Amy Graham, Quincy-Robyn Young

**Affiliations:** 1 Healthy Heart Program, Providence Health Care, St. Paul’s Hospital, 1081 Burrard Street, Vancouver B.C. V6Z 1Y6, Canada; 2 Department of Pathology and Laboratory Medicine, University of British Columbia, Vancouver, BC, Canada

**Keywords:** Smoking, Non-smoking, Risk factors, Varenicline, Interactive voice response

## Abstract

**Background:**

There is a significant resumption of smoking following smoking cessation using varenicline. Both smoking cessation medications and counseling have been shown to increase smoking quit rates at one year. Thus, the combination of varenicline and interactive voice response (IVR) telephony followed by extended IVR may further improve smoking cessation rates at one and two years.

**Methods:**

101 participants were recruited from the community via newspaper advertisement. They attended a group counseling session and were given smoking information booklets from the Canadian Cancer Society.

After 12 weeks of varenicline and 9 IVR calls, all participants who had quit smoking were randomized into 2 groups matched by levels of motivation and addiction as per baseline questionnaire score. The intervention group continued to receive bi-weekly IVR support for weeks 13 – 52. The control group no longer received IVR. The primary end-point was self-reported abstinence and exhaled carbon monoxide levels of less than 10 ppm for weeks 12, 52 and 2 years. Data were analyzed by Fisher’s exact test or Wilcoxon rank-sum test.

**Results:**

Of the 101 participants, 44 (43%) had stopped smoking after 12 weeks of varenicline and 9 IVR calls. Of these, 23 (52%) were randomized to receive IVR calls from weeks 13 to 52.

At 52 weeks, 26 (59%) participants remained smoke-free. Of the 23 with IVR, 12 (52.2%) stopped smoking compared to 14 of 21 (66.7%) without IVR. At 2 years, 40 of the 44 (90.9%) randomized participants were contacted and 24 of the 44 (54.5%) came in for testing. Fourteen (13% of the original cohort, 30% who were abstinent at 12 weeks and 53% who were abstinent at 52 weeks) remained smoke-free. Five of the 23 (21.7%) randomized to IVR and 9 of the 21 (42.9%) randomized to no IVR remained smoke-free at 2 years.

**Conclusions:**

In this pilot study of an apparently healthy population, extended IVR did not affect abstinence rates. There was no relapse prevention benefit in offering 9 months of continued IVR to subjects who had stopped smoking after receiving 3 months of varenicline and IVR treatment.

**Trial registration:**

ClinicalTrial.gov: NCT00832806

## Background

When administered for 12 weeks, the smoking cessation drug varenicline has been shown to lead to continuous abstinence rates of about 44% during the last 4 weeks of treatment and 22 – 23% at a one-year follow-up [1,2]. Although varenicline’s effect exceeded that of other drugs such as sustained-release bupropion, the cessation rates are still low. While medications can be effective in reducing withdrawal symptoms and improving treatment outcomes, a combination of pharmacotherapy and behavioral counseling is more likely to increase abstinence rates [3].

IVR systems have been applied in a variety of medical settings and have been used successfully to assess patients at home after hospital discharge for adverse outcomes [4].

Three Canadian studies have explored the potential of IVR to follow smokers after discharge as part of a comprehensive hospital-based smoking intervention (the “Ottawa Model”), demonstrating that it is feasible to use IVR in this setting and suggesting an intervention that includes optional automated post-discharge follow-up may increase smoking cessation. However, the specific contribution of IVR to cessation rates was assessed only in a small pilot study limited to smokers admitted with myocardial infarction. The study showed a benefit of IVR that did not reach statistical significance. Also, cardiac patients have higher rates of smoking cessation after hospital discharge than a general population [4].

The automated IVR uses algorithms and computerized speech recognition to engage smokers on the telephone: it gathers information; provides reinforcing messages and triages them to a study nurse for call-back within 2 working days if either the smoker or the nurse identify that help is required. IVR is a low cost, high-yield way of contacting smokers when they would not ordinarily be contacted.

In our study everyone received a combination of medication and smoking cessation counseling (through the IVR and call-back), from a study nurse if needed. This combination approach gave all participants proven treatment and an equal opportunity to stop smoking. We hypothesized that IVR telephony may decrease the relapse rate after smoking cessation.

## Methods

### Ethics and study design

This investigator initiated study was sponsored by Pfizer Canada, producers of varenicline. The study was scrutinized internally at the Healthy Heart Program and also by scientists at Pfizer Canada and Pfizer United States of America. It was approved by the Providence Health Care Ethics Committee.

The study is a two-arm, randomized pilot study of 2 years duration to determine the effect of 9 months of extended IVR on the effectiveness of smoking cessation after an initial 3 months of varenicline and IVR treatment. The randomized component of the study included only those who successfully quit smoking at 12 weeks.

Varenicline has been approved by Health Canada and marketed since 2007. The IVR system includes the option of direct nurse-to-subject over the telephone counseling. Both the automated IVR system and the direct nurse-to-subject over the telephone contact are classified as counseling.

The IVR uses algorithms and speech recognition to collect information from participants and monitor symptoms. It provides encouragement and reinforcing smoking cessation messages. These regular messages may serve to further strengthen participants’ sense of self-efficacy in remaining smoke-free [5]. If the participant requests a call-back then a study nurse would call them and help them get back on track with smoking cessation.

Short counseling interventions have been found to be very effective. Callback counseling provides a flexible, cost-effective intervention for smoking cessation that can be provided by a centralized service for a large population. It appears to encourage a greater proportion of quit attempts and to reduce the rate of relapse among those quitting [6].

### Study population

101 participants were recruited from the community in response to an advertisement in a local newspaper. None had a history of cardiac or other chronic disease.

Inclusion criteria: smoking 35 or more cigarettes per week or 5 or more cigarettes per day for at least 2 years with no period of abstinence longer than 3 months.

Exclusion criteria: use of any smoking cessation drugs or nicotine replacement drugs in the last 3 months, use of medications to treat depression or any psychiatric illness, history of depression or an unstable medical condition.

Exclusion criteria were identified by self-report. The study investigators met with each participant to complete a checklist of inclusion and exclusion criteria as approved by the hospital ethics committee. None of the participants who attended the initial study information session had a chronic disease. Those who had experienced depression or had a mental health condition in the past year were excluded because of the varenicline warning label for agitation, hostility, depression or changes in behaviour or thinking. We excluded people who had experienced these symptoms in the past year.

All potential participants attended an information session about smoking, smoking cessation, varenicline, eligibility for the study, and the relevance of the questionnaire about demographics, motivation, stress, and smoking. They were given an information package including the Canadian Cancer Society booklets *For Smokers Who Don’t Want to Quit* and *For Smokers Who Want to Quit*.

All participants gave written informed consent.

The study was performed in the Healthy Heart Program, an outpatient clinic. The focus of the program is helping people minimize their risk factors for coronary artery disease.

### Initial varenicline and IVR treatment

Participants received a 12-week supply of varenicline: 0.5 mg to be taken on days 1–3, 0.5 mg twice a day on days 4–7, and 1 mg twice a day until the end of week 12. At the initial visit participants chose a target quit date between 8 and 14 days after starting varenicline and gave their preferred calling time for the IVR technology.

The IVR intervention consisted of 2 parts: establishing it is speaking to the study participant and the main data collection section. As instructed at the beginning of the call, the participant answers “yes” or “no” to all questions except when asked about their level of confidence and their side effects. The IVR asks if they have had a cigarette since their quit date, if they have smoked a cigarette, even a puff, if they have used varenicline in the last 14 days, have they experienced any side effects, how confident they are that they will remain a non-smoker, and would they like to have a study nurse call them to help prevent relapse or provide advice about varenicline. Finally, there is a positive reinforcing message thanking and congratulating them followed by “remaining smoke-free is the single most important thing you can do for your health”. The calls are 3–5 minutes long, depending on their answers and which part of the algorithm they are directed to. The IVR made a call on their quit day, then on day 3, 8, and 11, and every 2 weeks thereafter.

After the 12th week of treatment, those who had stopped smoking were asked to come in for an exhaled carbon monoxide level measurement to confirm their non-smoking status. The primary end-point was self-reported abstinence and exhaled carbon monoxide levels of less than 10 parts per million (ppm) at 12 and 52 weeks and 2 years.

### Intervention and control cohorts

Participants who had quit smoking at 12 weeks were randomized into 2 groups matched by their level of motivation and level of addiction as per psychometric questionnaire at baseline. This was a stratified randomization whereby participants were categorized by motivation and addiction. The intervention group continued to receive IVR calls every 2 weeks from weeks 13 – 52. The control group did not receive further IVR.

At 52 weeks, all participants were asked to come in for a follow-up appointment and complete the same questionnaire administered at the initial baseline visit. Weight and waist circumference were measured and in those who had stopped smoking an exhaled carbon monoxide level was measured.

At 2 years all participants were asked to come in for a final visit to measure weight and waist circumference and determine smoking status. For those who were not smoking an exhaled carbon monoxide level was measured.

### Analysis

To date, a single all-encompassing questionnaire to assess level of motivation to stop smoking has not been developed. Rather, it is agreed that assessment of nicotine dependence, stage of change, balance of pros and cons, level of temptation, confidence to stop smoking, and level of stress and mood is best used in combination to assess motivation [7-9].

Questionnaire data were collected at the initial and 52 week visit. The Stage of Change algorithm was used to determine the stage of readiness for change [10]. Pros and cons of change were assessed using the Decisional Balance Scale [11]. This measured participants’ perceived benefits and drawbacks of stopping smoking. Their self-efficacy was measured on the Self-Efficacy and Temptation Scale [12]. These questionnaires have been used in the Canadian Heart and Stroke Foundation funded trial – *The Multicentre Biofeedback Reactivity Trial: Modifying Cardiovascular Reactivity to Stress During the Acute Phase of Smoking Cessation*[13].

The *Fagerstrom Tolerance Test of Nicotine Dependence* was used to determine level of dependence on nicotine and *The Stop D Scale* (Stress and Mood) to provide severity scores for each of the following areas: depression, anxiety, stress, anger, and poor social support [14,15]. The answers to the questionnaire provide information about the phenotype – who responds to varenicline and IVR treatment and who does not.

Motivation is a complex subject that involves motives, intents, values, and probability of success [16]. Motivation is considered critical to changing problem behaviours and to engaging in health-protection behaviours such as stopping smoking. Motivation for change typically refers to both reasons for change and the strength of the desire or commitment to make the change [17].

Age, gender, income, social support and number of cigarettes smoked per day are among the factors that have been shown to influence ability to stop smoking [18,19]. Table 1 summarizes selected variable frequencies that were included in the questionnaires. These are the variables with the strongest trend toward significance.

**Table 1:** Summary of selected variable frequencies

**Variable**	**All subjects (n = 101)**
Sex	
Missing	1 (.%)
Male	67 (67.0%)
Female	33 (33.0%)
Present living status	
Married	45 (44.6%)
Cohabiting	9 (8.9%)
Coupled and not cohabiting	1 (1.0%)
Widowed	4 (4.0%)
Separated	3 (3.0%)
Divorced	13 (12.9%)
Single	26 (25.7%)
Age at baseline	
Median (IQR)	54.0 (45.0, 62.0)
Mean (SD)	52.6 (11.8)
Min & max	(25.0, 73.0)
Weight at baseline (kg)	
Median (IQR)	76.4 (66.6, 90.7)
Mean (SD)	78.8 (16.6)
Min & max	(48.4, 136.0)
Waist at baseline (cm)	
Median (IQR)	91.0 (83.0, 100.0)
Mean (SD)	92.8 (13.9)
Min & max	(44.5, 134.0)
Number of cigs. smoked per day at baseline	
Missing data	1
Median (IQR)	18.0 (13.0, 22.0)
Mean (SD)	19.2 (9.8)
Min & max	(6.0, 55.0)
Status at week 12	
Still smoking	57 (56.4%)
Not smoking	44 (43.6%)
Status at week 52	
Missing data	33 (.%)
Smoking	40 (58.8%)
Not smoking	27 (39.7%)
Died	1 (1.5%)
Randomized to extended IVR	
No	21 (47.7%)
Yes	23 (52.3%)

### Statistical analysis

Summary statistics were used to describe the demographic characteristics of the study participants. The univariate relationship between the outcome and the potential predictors were analyzed by Fisher’s exact test or Wilcoxon rank-sum test as appropriate.

## Results

Of the 101 participants, 44 (43%) had stopped smoking after 12 weeks of varenicline and 9 IVR calls. Of these 44 subjects, 23 (52%) were randomized to receive extended IVR calls from weeks 13 to 52 and 21 (48%) were in the control group. Table 2 provides the selected variable frequencies in subjects eligible for randomization at week 12 (n = 44).

**Table 2:** Subjects eligible for randomization at week 12 (N = 44)

**Variable**	**Control (n = 21)**	**Extended IVR (n = 23)**
Sex		
Male	14 (66.7%)	13 (56.5%)
Female	7 (33.3%)	10 (43.5%)
Present living status		
Married	7 (33.3%)	12 (52.2%)
Cohabiting	1 (4.8%)	2 (8.7%)
Coupled and not cohabiting	0 (0.0%)	1 (4.3%)
Widowed	2 (9.5%)	2 (8.7%)
Separated	1 (4.8%)	0 (0.0%)
Divorced	3 (14.3%)	3 (13.0%)
Single	7 (33.3%)	3 (13.0%)
Age at baseline		
Median (IQR)	57.0 (48.0, 61.0)	58.0 (50.0, 64.0)
Mean (SD)	54.2 (10.0)	55.7 (10.9)
Min & max	(30.0, 69.0)	(27.0, 73.0)
Weight at baseline (kg)		
Median (IQR)	72.5 (64.4, 84.0)	76.4 (67.7, 98.3)
Mean (SD)	74.7 (13.6)	83.3 (21.6)
Min & max	(55.1, 105.7)	(48.4, 136.0)
Waist at baseline (cm)		
Median (IQR)	91.0 (83.0, 98.0)	95.0 (87.0, 106.0)
Mean (SD)	90.7 (11.4)	98.4 (15.4)
Min & max	(71.0, 117.5)	(79.0, 134.0)
Number of cigs. smoked per day at baseline		
Median (IQR)	16.0 (10.0, 20.0)	18.0 (13.0, 22.0)
Mean (SD)	17.3 (8.6)	18.5 (6.6)
Min & max	(6.0, 40.0)	(7.0, 37.0)

Of the 44 participants who had quit smoking at 12 weeks, 26 (59%) remained smoke-free at 52 weeks, 12 of these had received extended IVR and 14 were in the control group (12/23 (52%) vs. 14/21 (66.7%); p = 0.33). Table 3 provides the status at 52 weeks for those eligible for randomization at week 12 (n = 44).

**Table 3:** Status at 52 weeks for those eligible for randomization at week 12 (N = 44)

**Outcome**	**Overall (N = 44)**	**Control (N = 21)**	**Extended IVR (N = 23)**	**p-value**^**1**^
Status at week 52				
Missing	4	2	2	0.333
Smoking	14 (35.0%)	5 (26.3%)	9 (42.9%)	
Not smoking	26 (65.0%)	14 (73.7%)	12 (57.1%)	

^1^Based on Fisher’s exact test.

One participant who was not randomized stopped smoking after surgery for lung cancer. As a result, there were 27 confirmed non-smokers at 52 weeks but only 26 had been randomized at 12 weeks. One participant was diagnosed with lymphoma, randomized to receive extended IVR and remained a non-smoker at 52 weeks, and one participant who failed to quit at 12 weeks, died from an AIDS related illness.

**Figure 1:**
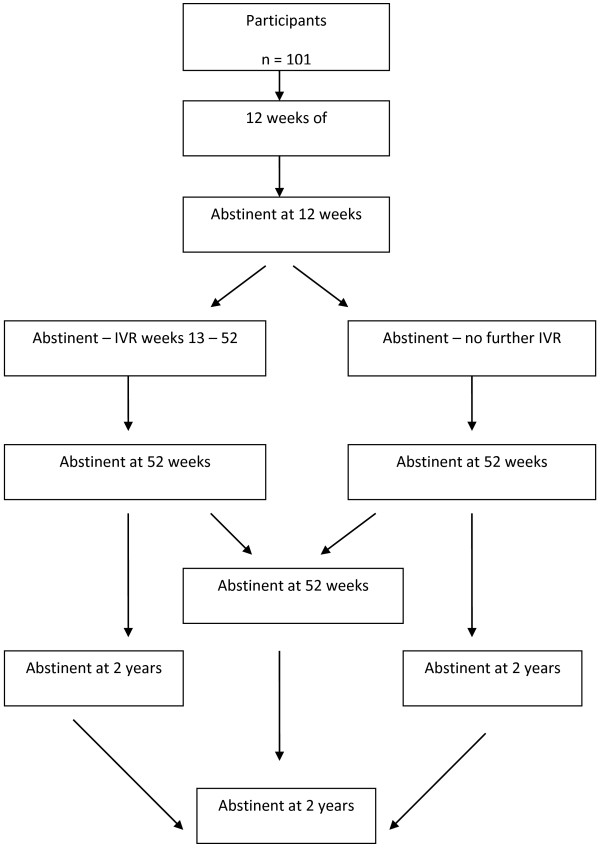
Flow chart.

At 2 years, 40 (39%) of the original 101 participants were contacted and 24 (23%) had their carbon monoxide measured. There were 14 (13% of the study population, 30% of those abstinent at 12 weeks, 53% of those abstinent at 52 weeks) who were confirmed non-smokers. Of these, 5 (21% of those abstinent at 12 weeks) had received extended IVR. Thus, 5 of 23 (21.7%) on IVR versus 9 of 21 (42.9%) on no IVR (chi-square = 2.26, p = 0.13) remained smoke-free at two years. Figure 1 displays a summary flowchart of the study results at 12 weeks, 1 and 2 years.

The IVR responses did identify those who were struggling to stop smoking and these were the participants that were called back. There were no common psychosocial or demographic characteristics, as identified in the questionnaire, that indicated who would respond to the IVR calls and who would not.

For participants in the extended IVR group, week 12 – 52, there was a low percentage of IVR calls completed (mean 37%, median 34%) and a lower percentage of assessments completed (mean 16%, median 16%). These were the participants who had stopped smoking at 12 weeks and were the subject of investigation.

### Variables predicting smoking cessation

Our study participants were recruited from the community, while many studies included recently hospitalized subjects who may have a greater degree of motivation to stop smoking.

Income over $54,000. showed a trend toward improved smoking cessation rates but was not statistically significant.

Table 4 displays a summary of the association between potential predictors and smoking status at week 12. Table 5 displays a summary of these potential predictors at week 52.

**Table 4:** Association between potential predictors and smoking status at week 12 univariate analysis

**Variable**	**Smoking (n = 57)**	**Not smoking (n = 44)**	**p-value**^**1**^
Sex			0.392
Missing	1 (.%)	0 (.%)	
Male	40 (59.7%)	27 (40.3%)	
Female	16 (48.5%)	17 (51.5%)	
Present living status			0.554
Married/Cohabiting	32 (59.3%)	22 (40.7%)	
Single/Others^2^	25 (53.2%)	22 (46.8%)	
Age at baseline			0.104
Median (IQR)	52.0 (40.0, 61.0)	57.5 (49.0, 63.0)	
Mean (SD)	50.8 (12.6)	55.0 (10.4)	
Min & max	(25.0, 70.0)	(27.0, 73.0)	
Weight at baseline (kg)			0.856
Median (IQR)	77.2 (66.7, 90.7)	75.4 (64.8, 90.2)	
Mean (SD)	78.5 (15.0)	79.2 (18.5)	
Min & max	(50.2, 109.5)	(48.4, 136.0)	
Waist at baseline (cm)			0.319
Median (IQR)	91.0 (82.5, 99.0)	92.3 (85.3, 101.5)	
Mean (SD)	91.3 (13.7)	94.7 (14.0)	
Min & max	(44.5, 127.0)	(71.0, 134.0)	
Number of cigs. smoked per day at baseline			0.467
Median (IQR)	20.0 (14.0, 23.0)	18.0 (13.0, 20.0)	
Mean (SD)	20.2 (11.2)	18.0 (7.6)	
Min & max	(6.0, 55.0)	(6.0, 40.0)	

^1^Depending on the type of the variable, p-value is based on Fisher’s exact test or Wilcoxon rank-sum test.

^2^Others include widowed, separated, divorced and coupled (not cohabiting).

**Table 5:** Association between potential predictors and smoking status at week 52 - for those randomized

**Variable**	**Smoking (n = 14)**	**Not smoking (n = 26)**	**p-value**^**1**^
Treatment group			0 .333
Control	5 (26.3%)	14 (73.7%)	
Extended IVR	9 (42.9%)	12 (57.1%)	
Sex			0.736
Male	8 (32.0%)	17 (68.0%)	
Female	6 (40.0%)	9 (60.0%)	
Present living status			0.510
Married/Cohabiting	6 (28.6%)	15 (71.4%)	
Single/Others^2^	8 (42.1%)	11 (57.9%)	
Age at baseline			0.966
Median (IQR)	57.5 (50.0, 63.0)	59.0 (48.0, 64.0)	
Mean (SD)	56.3 (8.5)	55.3 (11.8)	
Min & max	(41.0, 69.0)	(27.0, 73.0)	
Weight at baseline (kg)			0.092
Median (IQR)	69.7 (64.2, 77.4)	80.9 (68.1, 91.5)	
Mean (SD)	72.9 (15.4)	82.4 (19.8)	
Min & max	(55.1, 111.0)	(48.4, 136.0)	
Waist at baseline (cm)			0.072
Median (IQR)	87.8 (83.0, 95.0)	96.5 (89.0, 103.5)	
Mean (SD)	90.1 (13.6)	97.3 (13.9)	
Min & max	(71.0, 125.5)	(72.5, 134.0)	
Number of cigs. smoked per day at baseline			0.493
Median (IQR)	19.0 (12.0, 25.0)	17.0 (13.0, 20.0)	
Mean (SD)	18.9 (8.4)	16.9 (7.3)	
Min & max	(7.0, 37.0)	(6.0, 40.0)	

^1^Depending on the type of the variable, p-value is based on Fisher’s exact test or Wilcoxon rank-sum test.

^2^Others include widowed, separated, divorced and coupled (not cohabiting).

## Discussion

The study had a unique design and a long (2 year) follow-up.

The major appeals of using IVR are its high level of accessibility, and cost-effectiveness. Telephones are simple to use and familiar to people of most demographics, an advantage of IVR over some of the similar internet-based approaches that are currently being employed [5].

Research has shown that levels of IVR compliance tends to be relatively high when used for symptom monitoring and as an adjunct to the treatment of chronic pain [5]. This may indicate that IVR technology works well for people when they feel that it is beneficial and they are motivated to reach an outcome such as pain management.

Cardiac patients also have higher rates of smoking cessation after hospital discharge than a general population [4]. A study from The Ottawa Heart Institute identified almost 1300 patients at admission and 91% received intervention to help them quit smoking. Six months after discharge 44% of cardiac patients were not smoking after 10 weeks of nicotine patch therapy and 3 IVR calls [20].

A study with a general population showed after 12 weeks of varenicline or 10 weeks of transdermal nicotine patch, the last 4 weeks of treatment showed a significantly greater abstinence rate with varenicline (55.9%) than transdermal nicotine patch (43.2%; OR 1.70, 95% CI 1.26 to 2.28, p < 0.001). At 52 weeks these numbers decreased to 26.1% for varenicline and 20.3% for transdermal nicotine patch (OR 1.40, 95% CI 0.99 to 1.99, p = 0.056) [21].

At 3 months this 55.9% quit rate with varenicline compares with our 43% quit rate after 3 months of vareniclince and IVR. At 52 weeks the 26.1% quit rate compares with 25% of our original group (n = 26 out of 101). The quit rates for dual therapy varenicline and IVR were lower at 3 months in our study than for monotherapy varenicline.

While our study had a small sample size, it was interesting that those who were not smoking at 12 weeks and received extended IVR from weeks 13 – 52 had a 52.5% quit rate at 52 weeks. Those who were not smoking at 12 weeks and did not receive extended IVR had a 66.7% quit rate. It would appear that if you are able to quit by 12 weeks with varenicline and IVR you have a higher likelihood of not smoking at 52 weeks than if you had stopped with use of varenicline alone.

### Relapse prevention

The Cochrane Review for “relapse prevention interventions for smoking cessation” found that there is insufficient evidence, at the moment, to support the use of any specific behavioural intervention for helping smokers who have successfully quit for a short time to avoid relapse. The verdict is strongest for interventions focusing on identifying and resolving tempting situations, as most studies were concerned with these. There is little research available regarding other behavioural approaches. However, extended treatment with varenicline (past 12 weeks) may prevent relapse [22].

All participants in the study consented to receiving IVR calls. As IVR and varenicline were the basis of the study protocol it is difficult to determine how many were motivated to participate in IVR contact. There were no predictors (age, gender, number of cigarettes smoked per day) at 12 and 52 weeks as to who would respond to the IVR calls. Also, for those who had quit smoking at 12 weeks and were randomized to receive 19 additional calls between week 12 and 52, there were no predictors.

The IVR initiates contact as opposed to Quit Lines that people call. If participants request a call back or if their IVR responses indicate they are struggling with remaining smoke-free, are smoking, or experiencing side effects, a study nurse calls them. Proactive telephone counseling not initiated by calls to helplines helps smokers interested in quitting (44 studies, >24,000 participants, RR 1.29, 95% CI 1.20 to 1.38) [4].

There is some evidence of a dose response; one or two brief calls are less likely to provide a measurable benefit. Three or more calls increase the chances of quitting compared to a minimal intervention such as providing standard self-help materials, brief advice, or compared to pharmacotherapy alone. Telephone quitlines provide an important route of access to support for smokers, and call-back counseling enhances their usefulness [23].

In our study, participants received 9 IVR calls in the first 12 weeks and those who were randomized to receive extended IVR from weeks 13 – 52 received an additional 19 calls. We are not aware of any studies with this many IVR calls for this long of a period.

While relapse was progressive from week 13 through 52, those who had quit with IVR and varenicline at 12 weeks had relatively low relapse rates for smoking cessation by 52 weeks. Interestingly, those who had quit at 12 weeks (43%) and went on to receive extended IVR had a 52.5% quit rate at 52 weeks (n = 12/23). Those who had quit at 12 weeks and did not receive further care had a 66.7% quit rate (n = 14/21).

Future studies will hopefully address the possibility of an inverse benefit ratio of, for example, receiving more than 9 IVR calls. To our knowledge, there have been no published trials providing IVR and telephone call-back counseling as an adjunct to varenicline for smoking cessation.

We were not able to follow-up with study subjects to find out why they lost interest with the IVR.

The time of year chosen for follow-up may not have been conducive to attending follow-up appointments and responding to IVR calls. Follow-up appointments were scheduled at 12 weeks, 52 weeks and 2 years. All of these times coincided with summer season which may have been a barrier to attending. Also, the opportunity to receive the varenicline at no charge may have attracted a select population. The study participants were a lower income group ($42,000. average annual income) than the provincial average ($67,000). Other studies have shown a link between lower average income and ability to stop smoking [24,25].

## Conclusions

Our data show a lack of significant reduction in relapse rates at 52 weeks in apparently healthy participants who had received extended IVR (weeks 12 – 52) after stopping smoking with an initial 12 week treatment of varenicline and IVR.

Although age, treatment, baseline weight and income showed trends as predictors of smoking cessation, they were not statistically significant. These findings suggest a larger study is needed before allocating health care funds for IVR in a healthy population without chronic disease.

## Competing interests

Jiri Frohlich was a member of Pfizer (Canada) Medical Advisory Board and received speaking honoraria. He also participated in several clinical trials and received grants for investigator initiated studies.

## Authors’ contributions

JF obtained research funding and supervised all intervention development. JF and BM participated in the conception of the study and developed its design. AG and BM developed interactive voice response telephony interview scripts and analyzed and interpreted its data. QY provided questionnaire development and analysis methods. BM prepared data for analysis, including quality assurance and data consolidation. BM wrote the manuscript draft and JF revised it critically for intellectual content. All authors read and approved the final manuscript.

## Pre-publication history

The pre-publication history for this paper can be accessed here:

http://www.biomedcentral.com/1471-2458/13/824/prepub
